# Green-Synthesized Nanomaterials for Catalytic Reduction of para-Nitrophenol and Methylene Blue: Recent Advances and Perspectives

**DOI:** 10.3390/nano16060362

**Published:** 2026-03-16

**Authors:** Himanshi Soni, Monika Bhattu, Mikhael Bechelany, Jagpreet Singh

**Affiliations:** 1 Centre of Research Impact and Outcome, Chitkara University, Rajpura 140417, Punjab, India; 2 Bahra Research Innovation & Knowledge Cluster, Rayat Bahra University, Greater Mohali 140103, Punjab, India; 3 Institut Européen des Membranes (IEM), UMR-5635, University of Montpellier, ENSCM, CNRS, Place Eugène Bataillon, CEDEX 5, 34095 Montpellier, France

**Keywords:** catalyst, green synthesis, methylene blue, nanomaterials, nitrophenol, SDGs

## Abstract

Nitrophenol (NP) and methylene blue (MB) are considered among the most hazardous organic contaminants frequently released from pharmaceutical, textile, and paper industries, posing significant risks to both human health and the environment. The conventional treatment involves adsorption, oxidation, biological, filtration, and other photochemical degradation methods, which often suffer from low efficiency, limited reusability, and the production of secondary toxic by-products. In this context, the nanomaterials (NMs) mediated catalytic reduction of MB into leucomethylene blue and p-NP into p-aminophenol (p-AP) has emerged as a promising approach, due to its high efficiency and effectiveness. This review emphasizes the green synthesis of NMs for catalytic applications, which align with the principles of the circular economy and the Sustainable Development Goals (SDGs). This thorough review systematically examines the mechanistic understanding of the reduction of both p-NP and MB via different green synthesized NMs and evaluating their catalytic efficiencies. Furthermore, a detailed discussion of the reduction of pollutants (p-NP and MB) is provided, along with their mechanistic insights. In addition, this paper also provides a comparative table highlighting the effects of using different precursors, experimental conditions on the conversion catalytic efficiency and reusability potency. Thus, this work provides the insights into recent research on the catalytic reduction of p-NP and MB into valuable products, highlighting the significance of green synthesized nanocatalysts for effective wastewater treatment.

## 1. Introduction

In recent years, industrialization and continuously increasing urban growth have led to the heavy discharge of toxic contaminants into the environment [1,2,3]. These toxic contaminants significantly affect the ecosystem and environmental quality. Among the large number of contaminants in the environment, nitroaromatic compounds and synthetic dyes are of particular concern due to their high toxicity and chemical stability [4,5,6,7]. These contaminants persist in the environment for a longer time. Nitrophenol is an aromatic nitro compound that is utilized as an intermediate in pharmaceuticals and pesticides [6]. On the other hand, MB is a well-known synthetic cationic dye widely utilized in the textile and paper industries [7,8]. These compounds are continuously used in industries, and without proper disposal, they are discharged into the environment, which affects both water and air quality. Excessive commercial use of MB and p-NP generates toxic effluent, which poses substantial threats to ecosystems and human health [9]. Therefore, it is necessary to identify effective approaches to remediate the environment. Conventional wastewater treatment techniques include physical methods such as ion exchange, filtration, and adsorption [10,11]. Chemical methods involve coagulation, precipitation, oxidation processes, and electrochemical treatments [12,13]. On the other hand, biological methods include anaerobic digestion, sludge processes, and phytoremediation. However, these processes have their own drawbacks that limit their applicability. These methods have limited efficiency, high operational and maintenance costs, and they also include formation of toxic by-products, excessive use of toxic reagents, and in some cases slow reaction rates. Catalysis has been shown to be a viable alternative for the elimination of p-NP and MB [14]. The catalytic process can be used to boost the degradation rate of p-NP into safer substances and enable effective breakdown of MB into less toxic. colorless products, overcoming the limits of conventional approaches. Furthermore, catalysis helps to mitigate the negative environmental effects of these contaminants, notably in wastewater treatment systems. Traditionally, numerous organic catalysts such as activated carbon, conductive polymers, enzymes, and graphene oxide have been used. In addition, inorganic catalysts, including clay minerals, zeolites, and various metal oxides, have also been used to degrade p-NP and MB. Despite their advantages, these materials present several limitations. As we know, zeolites possess a low number of active sites, which decreases the efficiency of the catalyst. Similarly, clay minerals have a large particle size, which lowers the surface area, while metal oxides favor undesirable reaction pathways.

To address these issues, nanomaterials (NMs) have been introduced to promote the effective removal of MB and p-NP. NMs have been considered as the best alternative owing to their small size, high surface area, ease of functionalization, and tunable properties [14,15]. Initially, synthesis methods such as pyrolysis [16], solvothermal [17], sol–gel processes [17], sputtering [18], vapor-phase techniques [19], ultrasonic irradiation [20] and sonochemical synthesis methods have been employed [21]. However, these approaches often require (i) high operational cost, (ii) high energy consumption, and (iii) the utilization of toxic chemicals. Therefore, in the current scenario, researchers are working on the development of green routes for the fabrication of NMs that can be employed for the reduction of p-NP and MB. Green-synthesized NMs have been used for the catalytic reduction of p-NP and MB. These NMs are classified based on their composition and structure. This includes metal NPs (Ag, Au, Pd, Pt), metal-oxide NPs (TiO_2_, ZnO, Fe_3_O_4_), carbon-based NMs (carbon dots, graphene, graphene quantum dots), polymer-based NMs, magnetic NMs, and other low-dimensional materials. These NMs have been extensively studied as catalysts due to their distinctive properties [22,23,24,25,26,27]. Additionally, green NMs exhibit high catalytic activity due to their high surface-to-volume ratio, which enhances the availability of active sites on the surface of NMs. In the case of p-NP, electron migration from the reducing agent to the nitro group occurs via the catalyst surface, whereas MB reduction occurs through electron transfer to the dye’s chromophoric structure, resulting in the breakdown of the conjugated system and subsequent decolorization.

In lieu of above discussion , this review covers various NMs used as a catalyst for the reduction of p-NP and MB. To date, several review articles have been published that primarily focus on either specific categories of pollutants or particular classes of nanomaterials; however, such a fragmented approach often limits their comprehensive scope and restricts the accessibility of integrated knowledge for researchers seeking a broader understanding of the field.In contrast, the present review covers different categories of NMs, including carbon-based, polymer-based, metal, and metal-oxide NMs, for the reduction of both p-NP and p-AP, providing a better updated and unified platform for early-career researchersIn addition, a comparison table (Table 1) has been provided to highlight the novelty of this review in comparison with previously published reports, to the best of our knowledge. This paper comprises the discussion on background of p-NP and MB along with theirmolecular structures, and mechanisms involved in the removal of p-NP and MB using different green synthezied NMs. 

## 2. Background of MB and p-NP

**Figure 1:**
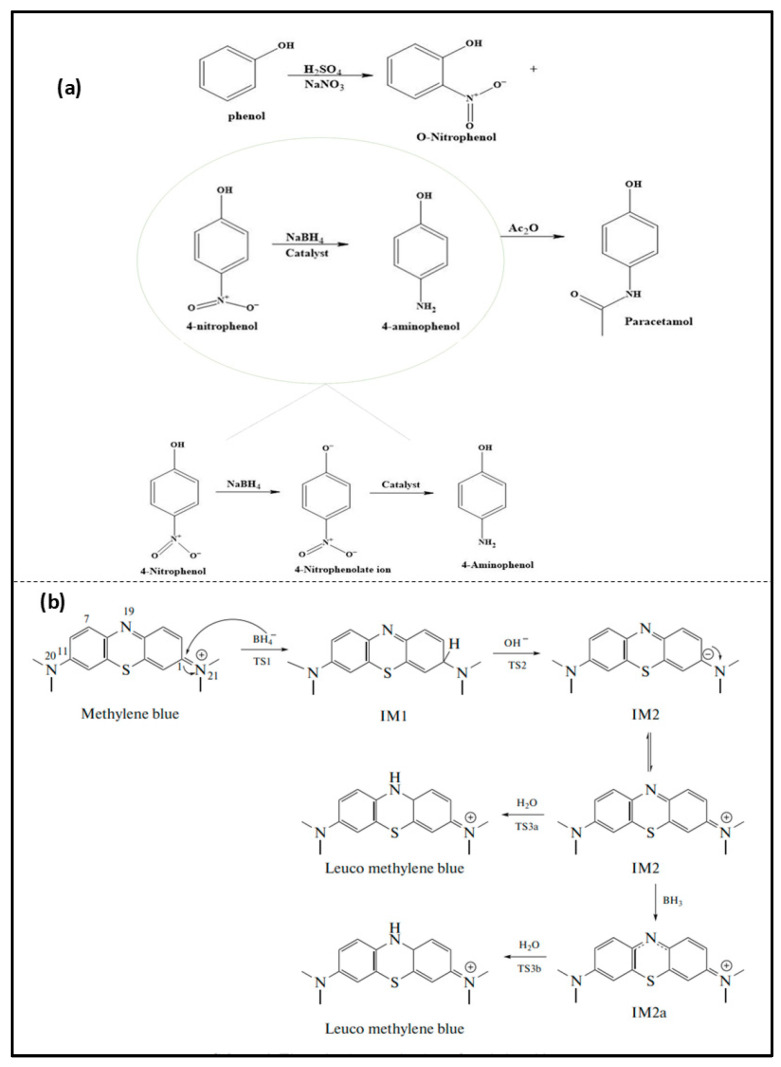
Mechanistic representation of (**a**) the catalytic reduction of nitrophenol; (**b**) the catalytic reduction of methylene blue [39].

Methylene blue (MB), with the chemical name of methylthioninium chloride (C_6_H_18_ClN_3_S), has been continuously utilized in the field of medicine ever since its discovery. In 1876, Heinrich Caro, a German chemist, first discovered MB as it grew from the dye industry in England [37]. Since it was discovered, it has worked as a synthetic drug used in medicine. After its discovery, it has been used in various fields of sciences and medicine [38]. MB is a synthetic cationic dye mainly discharged from the pharmaceutical, textile, and paper industries. It is also utilized in medical practice, research laboratories and hospitals. These pollutants are directly discharged into the environment after their application, which contaminates the aqueous environment. Owing to its extensive use, it is frequently released untreated into aquatic systems, posing risks due to its toxicity and long-term environmental exposure. Therefore, it is crucial to develop some strategies to convert it into less harmful products, thereby mitigating its harmful impact. On the other hand, p-NP, with molecular formula C_6_H_5_NO_3_, was discovered in the 19th century and is a toxic nitroaromatic compound that served as part of general studies on nitration reactions in the development of organic chemistry. The first scientist to introduce paracetamol was J. von Mering, whose work involved nitration and the conversion of the nitro group to paracetamol. NP is one of the toxic contaminants found in industrial wastewater that causes adverse impacts on the environment. Nitrophenol is involved in paracetamol formation, which is among the most commonly prescribed pain-reliever medications around the world. P-NP, ortho-nitrophenol, and metal nitrophenol are three isomers of nitrophenol. Among the three nitrophenol isomers, p-nitrophenol exhibits the fastest reduction rate. The reduced spatial interference between these functional groups facilitates more efficient electron transfer, resulting in a higher reduction rate compared to the ortho- and meta-isomers. p-NP is mainly discharged from pesticide industries, dye industries, and pharmaceutical industries. Moreover, p-NP is used as a model compound in catalytic reduction studies due to its well-known reduction kinetics and its distinct absorption peak at 400 nm. Consequently, several approaches have been employed to effectively remove p-nitrophenol from aqueous environments. Sodium borohydride is one of the well-known reducing agents used to reduce nitrophenol to aminophenol. The schematic representation of the reduction of these two pollutants using sodium borohydride is demonstrated in Figure 1.

## 3. Different Types of Wastes and Synthesis Methods

**Figure 2:**
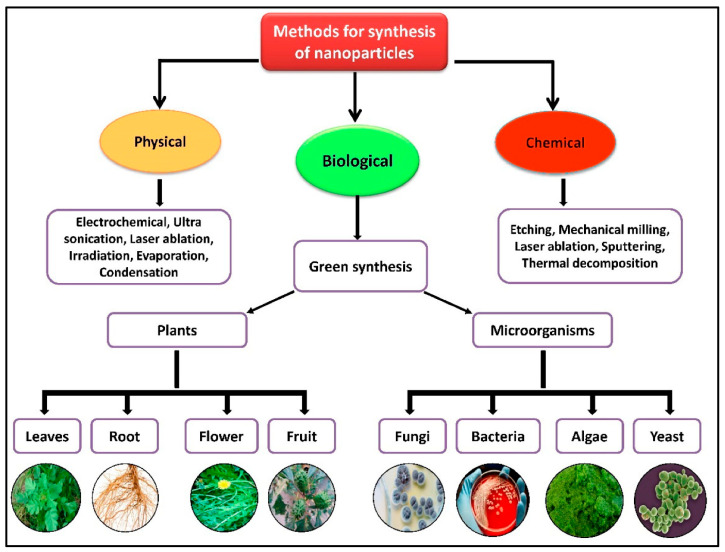
Flow chart representation of different synthesis methods of nanoparticles [43].

The increasing toxicity of chemicals and their hazardous impact on the environment and human health demand sustainable technology. Conventionally, the synthesis of NMs relies on the use of harmful chemicals, high energy consumption, high maintenance costs, and energy-intensive processes. To overcome such issues, current research is shifting towards the synthesis of NMs using green methods. The synthesis of NMs involves physical, chemical, and biological methods. The flow chart representation of all the synthesis methods involved is shown in Figure 2. Biological synthesis methods reduce the toxic impact of NMs on the environment by using environmentally friendly precursors. Green precursors involve plant extracts, enzymes, household waste, agricultural waste, and forest waste. Various studies have shown that plant extracts serve as reducing and stabilizing agents, thereby providing a good alternative to toxic chemicals. The main advantage of these precursors is that they are readily available and often inexpensive. Moreover, the utilization of biomolecules acts as a natural capping agent that improves stability and simultaneously prevents agglomeration. The green route for the preparation of NPs follows three steps: (i) collection and drying of precursors; (ii) fabrication of NMs; and (iii) centrifugation for separation. Numerous studies have reported the applicability of green precursors for the synthesis of NMs to decontaminate MB and p-NP. For instance, Muhammad Ismail et al. prepared a silver/taro power nanocomposite using taro rhizomes as a natural precursor for the conversion of p-NP. The study found that taro rhizome acts as a reducing agent for the removal of pollutants. In the study by Yao et al., gold NPs were extracted from e-waste for the reduction of nitrophenol [40]. The study by Cunha utilized coconut mesocarp sawdust [41]. In addition, Mansee et al. utilized glucose as a natural reductant for the conversion of MB [42]. Similarly, several studies have reported the usage of natural precursors for the synthesis of NPs acting as non-toxic catalysts.

## 4. Physicochemical Properties of Green-Derived Nanomaterials for the Reduction of MB and p-NP

The catalytic efficiency in the removal of pollutants like p-NP and MB relies on the physicochemical properties of the prepared nanomaterials. The presence of bioactive compounds, including polyphenols, flavonoids, amino groups, alkaloids, and others, acts as stabilizing and capping agents. This method replaces traditional processes that use harmful chemicals as stabilizers. Plant extracts are well-known rich sources of metabolites serving as both reducing and capping agents. Moreover, the synthesis of NMs using natural precursors depends on several aspects, including pH, temperature, contact time, and the type of precursor. Various studies reveal that the size and morphology of the prepared NMs depend on the type of precursor utilized during the synthesis process. An understanding of the physicochemical properties of green-derived NMs, including particle size, functional groups, band structure, crystallinity, and others, is important when observing the catalytic behavior of NMs towards MB and p-NP. A comparative table of the properties of green-prepared NMs, along with their ranges and roles in reduction, is discussed in Table 2.

## 5. Catalytic Reduction of MB and p-NP Using Green-Synthesized NMs

### 5.1. Catalytic Reduction of MB

MB is one of the well-known organic dyes utilized in pharmaceutical, textile, paper, and agricultural industries. The frequent discharge of pollutants from these industries causes severe stress on the environment, leading to contamination of air and water resources. MB persists in aquatic environments for a longer time due to its high chemical stability, strong oxidizing agents, and low biodegradability. Several studies have found that even at low concentrations, MB affects water quality by reducing light penetration, thereby harming photosynthetic processes. Exposure to this harmful pollutant causes respiratory disorders, skin diseases, eye diseases, and neurological diseases. Furthermore, incomplete degradation processes of MB result in the formation of harmful intermediates, raising concerns about environmental safety and human health. Table 3 highlights the physicochemical properties of methylene blue.

MB, chemically known as (3,7-bis(dimethylamino)-phenothiazin-5-ium chloride), involves a multi-step mechanism during its reduction. Studies reported in the past have used plant extracts and biowaste-derived NMs, including metal NPs, carbon-based nanostructures, metal oxides, and polymer-based NMs, for the efficient reduction of MB. Initially, (i) adsorption of MB molecules takes place on the surface of NMs; (ii) upon light irradiation, active charge carriers are generated; (iii) formation of ROS species occurs; (iv) reductive and oxidative breakage of the MB structure occurs; and (v) formation of non-toxic intermediates occurs. The rate of electron transfer and the formation of ROS species serve to promote the dye conversion process. Various studies have explored this phenomenon using different types of green-synthesized NMs to degrade MB. For instance, Hamad et al. prepared a CuNP/hydrochar nanocomposite for the catalytic reduction of methylene blue. The study utilized organic peel waste as a natural precursor for the preparation of the CuNP/HC nanocomposite via a hydrothermal method. The study exhibited a spherical morphology of CuNPs with sizes of 10–20 nm, as observed by TEM analysis. Complete degradation of MB was achieved within 10.27 and 11.73 min, with an average particle size of 1.6 nm. The prepared NPs showed a dual catalytic mechanism for the reduction of both p-NP and MB. In the case of the reduction of p-NP, the process involves activation of the borohydride ion and subsequent electron transfer through hydrochar. In contrast, the removal of MB occurred through electrostatic interactions, in which dye molecules are adsorbed onto the negatively charged hydrochar surface. In this study, hydrochar acted as an electron storage, while CuNPs acted as proton-transfer centers, enabling high stability of the prepared material. The degradation efficiency reached 100% for both pNP and MB under controlled optimized conditions. As per the Langmuir–Hinshelwood model, borohydride ions are initially adsorbed onto the surface of CuNPs, which subsequently dissociate to generate reactive electron–proton pairs. Simultaneously, *p*-NP molecules adsorb onto available sites on the catalyst through coordination via the nitro group. In the end, reduction proceeds via hydrogenation of the –NO_2_ group through nitroso (–NO) and hydroxylamine (–NHOH) intermediates, resulting in the formation of *p*-aminophenol, which then desorbs from the catalyst surface. The prepared catalyst maintained its stability for up to eight cycles for both p-NP and MB at pH 8 [9].

In another study, Lee et al. prepared gold NPs using Korean red ginseng root extract for the reduction of p-NP and methylene blue. The study reported that the synthesized AuNPs possessed an average size of 10.5 ± 2.4 nm ~ 13.9 ± 5.1 nm, indicating the formation of well-structured catalyst [44]. Similarly, Ravi et al. prepared iron oxide NPs using *Tinospora cordifolia* leaf extract to decontaminate water from MB. The study illustrated that Fe_3_O_4_-NPs achieved successful removal of MB with a removal rate of 88 ± 2.42% in 60 min. In addition, the prepared nanocomposite exhibited high stability, and the TC-Fe_3_O_4_-NPs could be reused for up to five consecutive cycles [45].

**Figure 3:**
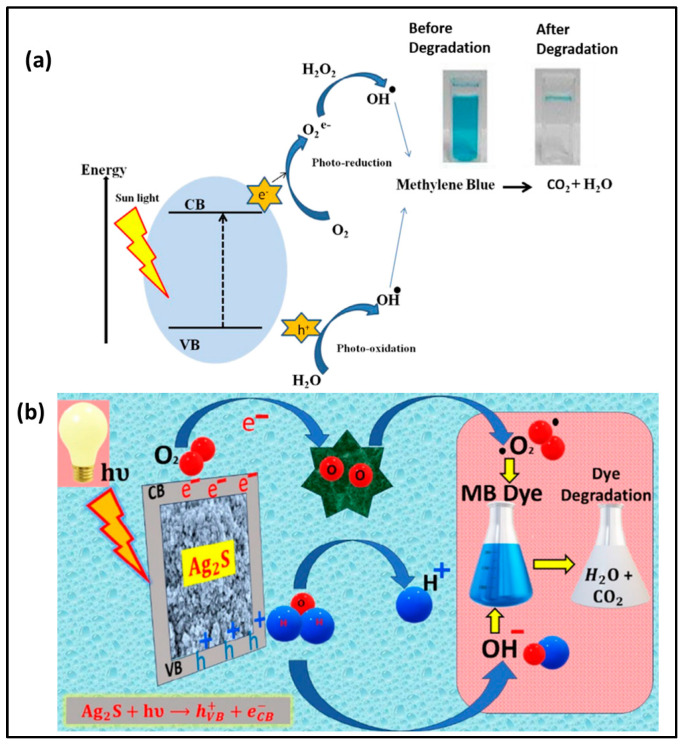
Mechanistic representation of the photocatalytic degradation of MB using green-synthesized (**a**) ZnO NPs [47]; (**b**) Ag2S NPs [46].

In addition, Mondal et al. prepared Ag_2_S NPs using lemon citrus as a natural precursor to convert MB into a less harmful product. The citric acid present in lemon citrus acted as an effective stabilizer for the synthesis of Ag_2_S NPs and prevented NP aggregation. In this study, two different catalyst dosages were prepared to evaluate the efficiency at different doses. It has been shown that 5 and 10 mg of Ag_2_S NPs were used for the degradation of MB dye to observe dose-dependent photocatalytic activity. The results demonstrated that 5 mg of catalyst achieved a maximum removal rate of 65%, whereas 10 mg of catalyst exhibited an enhanced removal efficiency of 85%. This was attributed to the availability of active sites, as smaller NPs offer a larger surface area and better electron–hole separation. Consequently, the Ag_2_S-20 sample acts as a good photocatalyst compared with others. Mechanistically, the study revealed that Ag_2_S NPs promote electron transfer rate from the VB to the CB, creating holes on the surface. The study reported that the particle size and surface defects play a main role in determining the efficiency of electron–hole separation. In addition, ROS species were found to contribute to the oxidative degradation of MB into CO_2_ and H_2_. MB molecules also undergo electron transfer to form MB^+^ species, which further participate in accelerated decomposition pathways as shown in Figure 3b. Moreover, the acidic nature of MB results in a weak negative surface charge, which influences electrostatic interactions and affects degradation efficiency [46].

Furthermore, Gupta et al. prepared ZnO NPs using *Abroma augusta* leaf extract for the degradation of MB. The synthesized NPs exhibited a size of 12 nm with a removal rate of 91%. The study revealed the importance of using *Abroma augusta* leaf extract in enhancing catalytic performance, as the removal efficiency was only 42% in its absence but increased up to 91% in its presence. AbA-ZnO plays a major role in accelerating the oxidation and reduction processes. During irradiation, electrons are generated in the valence band and holes in the conduction band. These photogenerated electrons move to the surface of the NPs. The surface of the NPs is filled with active sites for radical formation, where photogenerated holes react with water to generate OH radicals and further form superoxide species. These reactive radicals attack MB molecules and break their complex structure into less harmful products, as shown in Figure 3a [47].

The study by Bharathi et al. prepared AgNPs using *Merremia quinquefolia* (L.) Hallier f. leaf extract for the degradation of methylene blue. During the degradation process, the initial step involves the adsorption of MB molecules onto the surface of the photocatalyst due to electrostatic attraction and surface affinity. The photocatalyst absorbs photons with energies greater than its bandgap when it is exposed to light. The electrons jump from the VB to the CB, creating e^−^/h^+^ pairs. The photogenerated electrons and holes shift towards the surface of the catalyst, where electrons move to reduction sites and holes move to oxidation sites. At the surface of the catalyst, ROS species are formed, which further attack MB molecules, forming less stable intermediates. These intermediates undergo further oxidation, forming the end products. The study reported that the photocatalyst remains unchanged during the process and returns to its initial stage for reuse. Although some electrons and holes recombine, the catalyst regains its activity under continued light irradiation [48].

In addition, Saadi et al. prepared ZnO NPs for the reduction of methylene blue using a sol–gel process. The prepared NPs revealed a crystalline structure with an average size of 31.5 nm. Moreover, the degradation efficiency of methylene blue was reported to reach 72.3% after 7 h. In another study, Baqer et al. also prepared ZnO NPs for the reduction of MB dye using cinnamon extract and bay leaf extract. The study found a spherical morphology of ZnO NPs with an average size of 21.49 nm using cinnamon extract and 25.26 nm using bay leaf extract. Under solar light irradiation, the catalytic efficiency of ZnO NPs was monitored. The performance of the synthesized NPs varied according to surface area, size, and radiation source. The maximum degradation of methylene blue was achieved within 1 h. It was observed that with increasing irradiation time, the absorption peak of MB decreased continuously, and the dye was completely degraded within 1 h [49]. The study by Yildirim et al. reveals that, apart from metal-oxide NMs, bio-based composites have also exhibited promising results. In this study, the authors prepared the Russula delica/bentonite/tripolyphosphate (RDBNC) composite for the reduction of methylene blue and malachite green dyes. The degradation of MB is influenced by hydrogen bonding, π-π interactions, and electrostatic interactions. The adsorption process involved interactions between the surface of RDBNC and MB dyes. Furthermore, the process was strongly influenced by functional groups including hydroxyl, carboxyl, and amino groups. FTIR spectra revealed that C-O and Al-OH groups enhance the binding sites, and the shift in characteristic peaks confirmed the H-bonding contributions to dye degradation. Moreover, the prepared composite remained stable for up to four cycles without losing its adsorption capacity [50].

The study by Ubale et al. prepared Cr^3+^-doped nickel ferrite NPs using ginger extract. SEM analysis revealed the spherical morphology of the prepared NPs with sizes ranging from 22 to 27 nm. The study reported that ferrite NPs possess suitable bandgap energies, which make them effective for the catalytic conversion of MB. The photocatalytic degradation of MB was monitored under UV light. Complete degradation was achieved within 210 min, with the degradation efficiency reaching 78%–90% [51]. Similarly, Dhanalakshmi et al. prepared CuO NPs using the leaf extract of *Ceropegia deblis*. The bioactive compounds present in the extract acted as stabilizing and capping agents during NP formation. The prepared NPs exhibited spherical morphology with a size of 42.9 nm. Moreover, the degradation of methylene blue was reached within 105 min, with a 95.8% degradation rate [52].

### 5.2. Catalytic Reduction of p-NP Using Green-Synthesized NMs

The catalytic reduction of nitrophenol has gained significant attention from researchers as it is widely accepted as a model reaction to evaluate the efficiency of heterogeneous catalysts. In the past, various types of NMs have been used, including silica, metal–organic frameworks, carbon-based nanostructures, porous materials, and other inorganic materials, for the reduction of nitrophenol. These types of NMs possess various types of physical and chemical properties, such as small particle size, high surface area, and tunable surface functionality, which make them suitable for catalytic applications [28]. Table 4 highlights the physicochemical properties of p-Nitrophenol. The catalytic reduction of nitrophenol involves the use of a catalyst to accelerate the conversion of p-NP to p-AP. The most commonly utilized reducing agent during the conversion process is NaBH_4_.

For any type of catalytic material, two main factors are considered, namely reusability and high catalytic activity. The mechanism involved in the reduction of nitrophenol generally undergoes five main steps: (i) adsorption of NaBH_4_ onto the catalyst surface; (ii) conversion of NP into the nitrophenolate ion; (iii) dissociation of the borohydride ion to generate ROS; (iv) transfer of electrons from BH_4_^-^ions to the nitro group; and (v) formation of reaction intermediates. Conventional treatment methods, including chemical oxidation and adsorption, often exhibit low efficiency and may generate secondary toxic by-products. Therefore, to overcome these limitations, the use of green-prepared catalysts plays a main role in the reduction of nitrophenolic pollutants. In this context, Mahajan et al. (2025) [53] developed ZnO NPs using the leaf extract of Justicia adhatoda, which acts as a natural precursor for the degradation of NP. The as-synthesized NPs have a small crystalline size of 12.41 nm via green synthesis. The green precursor utilized here acts as both a reducing and stabilizing agent. Notably, the study achieved a degradation efficiency of 99.8% within 180 min under sunlight irradiation. This high degradation efficiency was attributed to the generation of a large number of ROS and efficient charge separation of photogenerated electron–hole pairs, supported by the high surface area of ZnO NPs. Furthermore, the conversion was found to be achieved within 15 min. Similarly, Elbayoumy et al. prepared CuO NPs for the reduction of p-NP to 4-AP using a crosslinked vinyl polymer. The prepared composite, employed as a heterogeneous catalyst, achieved conversion within 6 min [54].

**Figure 4:**
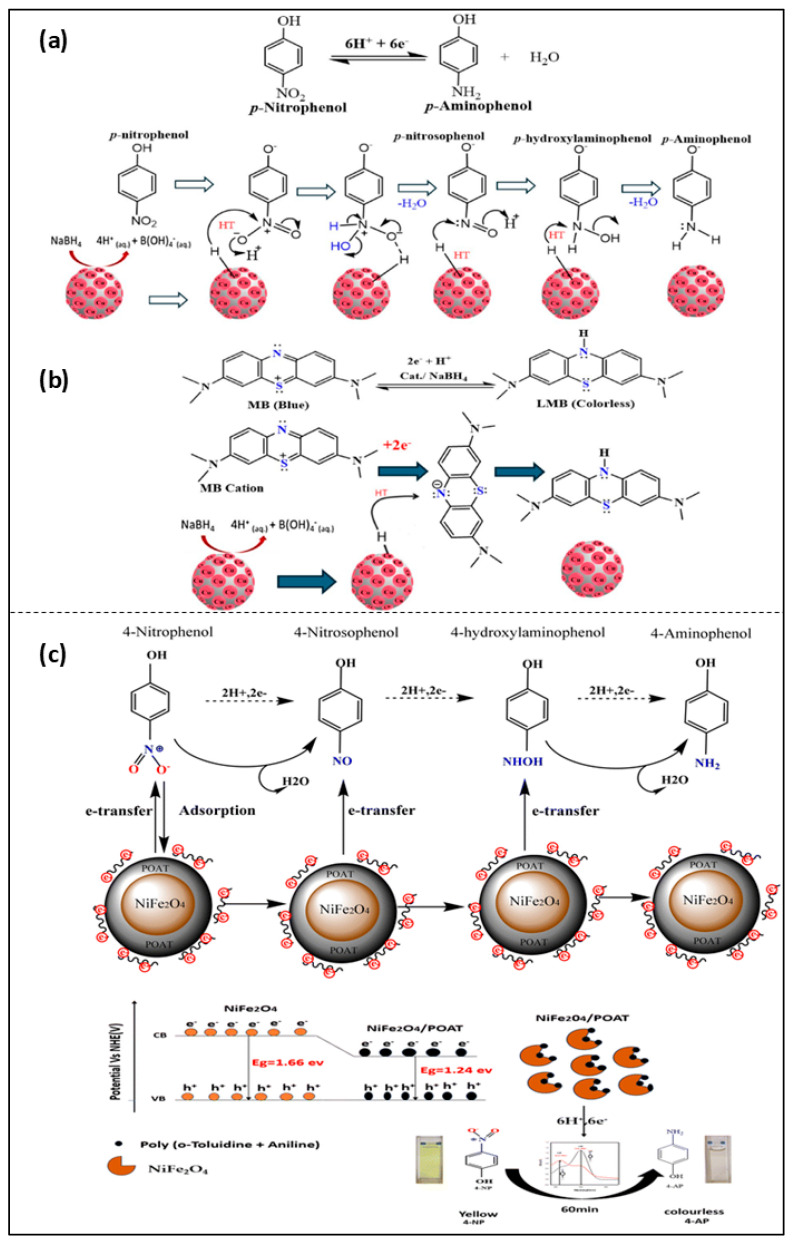
Schematic representation of the mechanism of (**a**,**b**) degradation of p-NP and MB using CuNP/hydrochar composite [9]; (**c**) reduction of p-NP using NiFe_2_O_4_/POAT [55].

Moreover, the study by Stambouli et al. prepared a NiFe_2_O_4_-based composite for the degradation of p-NP. The authors synthesized a (NiFe_2_O_4_)/(PAOT) nanocomposite, forming a stable structure with a size of 32–68 nm, as analyzed using techniques like FTIR, XRD, EDX, and SEM. The prepared nanocomposite exhibits magnetic properties and can therefore be easily recovered and reused for up to four cycles. The performance of the prepared catalyst for the degradation of p-NP was monitored under UV light irradiation using the Ni/PAOT composite. The conversion time was found to be 60 min with an efficiency of 99% at 15 ppm. The reduction was monitored through photon adsorption by NiFe_2_O_4_, due to its narrow bandgap, which generates electron–hole pairs, as shown in Figure 4c. The electrons from the conduction band are transferred to the nitro group of p-NP adsorbed on the catalyst surface. The -NO_2_ group is converted into nitroso and hydroxyamine intermediates and then reduced to an amine group, yielding aminophenol as the final product. The PAOT matrix utilized in the NC promotes charge separation and facilitates electron transport for efficient catalytic reduction [55]. Similarly, Hamad et al. prepared a CuNP/hydrochar nanocomposite to enhance the degradation of p-NP and methylene blue. The study utilized organic peel waste as a natural precursor for the preparation of the CuNP/HC nanocomposite via a hydrothermal method. TEM analysis revealed that CuNPs exhibit a spherical shape with sizes of 10–20 nm. The prepared NPs showed a dual catalytic mechanism for the conversion of both p-NP and MB. In the case of the reduction of p-NP, the process involves activation of the borohydride ion followed by electron transfer through hydrochar. The degradation efficiency reached 100% for both p-NP and MB under controlled, optimized conditions. Figure 4a shows that *p*-NP molecules adsorb onto the available active sites of the catalyst through coordination via the nitro group. In the end, reduction proceeds via hydrogenation of the –NO_2_ group through nitroso (–NO) and hydroxylamine (–NHOH) intermediates, resulting in the formation of *p*-aminophenol, which then desorbs from the catalyst surface. The prepared catalyst maintained its stability for up to eight cycles for both p-NP and MB at pH 8 [9].

Similarly, Ávila-Avilés et al. synthesized AuNPs and AgNPs from different types of tea, including black, green, red, and white tea. The extract of *camellia sinensis* has been used to prepare NPs, which are further used as a capping agent. The prepared NPs were reported to have sizes of 29.46 ± 53.92 nm for AuNPs and 33.11 ± 18.50 nm for AgNPs. The study found that different types of tea extracts affect the size, morphology, and degradation performance of the prepared catalysts. Functional groups, including flavonoids, polyphenols, amino acids, and proteins, have strong reducing and stabilizing properties and can donate electrons to metal ions. Furthermore, the catalytic reduction of AuNPs and AgNPs is strongly influenced by the presence of bioactive compounds in tea extracts. The reported reduction is monitored by UV–Visible spectroscopy through the formation of the nitrophenolate ion. The authors found that, in the absence of a catalyst, the reaction rate is too slow due to kinetic barriers. To overcome this issue, the prepared metal NPs were added, which transfer electrons between the borohydride ion and p-NP and thereby enhance the reduction rate [56]. Eisa et al. also prepared NiO NPs using cotton fiber under gamma radiation for the catalytic conversion of p-NP. The study prepared NiO NPs of spherical shape with an average size of 32 nm. These NPs were incorporated into a cotton matrix to form an NiO@cotton catalyst, which provided high efficiency. Sodium borohydride was employed as the reducing agent for the degradation of p-NP. The UV-Vis adsorption spectra confirmed degradation within 26 min. Moreover, the NiO@cotton demonstrated good stability for up to 10 cycles, with conversion rates of 88.3% and 84.9%, respectively. The authors reported that the slight decrease in catalytic activity was mainly due to the adsorption of by-products on the catalyst surface, which blocks access to active sites [57]. The study by Chan et al. utilized plastic waste for the synthesis of a catalyst using the cross-linking reaction of PET-derived oligos. The authors found the conversion of p-NP by observing the decrease in absorbance intensity of nitrophenol and the increase in absorbance intensity of p-aminophenol [58]. Shen et al. also prepared AuNPs from the extract of AS WL-Au for the degradation of p-NP. The study revealed that different functional groups on the catalyst surface promotes stability and selectivity. The as-prepared AuNPs acted as a catalyst for the degradation of p-NP, where a spectral shift from 317 to 400 nm was observed using a reducing agent to form the nitrophenolate ion. The complete conversion of p-AP from p-NP takes 2–6 min using AuNPs. Moreover, the authors reported that kinetic barriers cause differences in potential between donor and acceptor species. Electrons are transferred from hydride ions to the nitro group in the presence of AuNPs, resulting in the formation of 4-AP [59].

**Figure 5:**
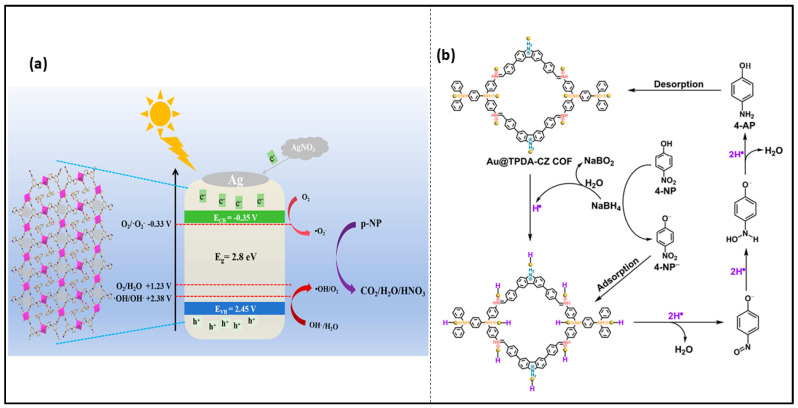
(**a**) Proposed photocatalytic reduction of p-NP using AgNO_3_ under light irradiation [60]; (**b**) Adsorption–reduction–desorption mechanism of p-NP using the Au@TPDA-CZ-COF catalyst [40].

Similarly, Yao et al. prepared AuNPs from e-waste and then incorporated them into TPDA. The Au@TPDA-CZ-COF composite significantly promoted the reduction rate of nitrophenol, resulting in a 99.24% conversion rate within 5 min. Further, the reusability performance reveals the stability of the prepared catalyst for up to five consecutive cycles. The reduction mechanism of the Au@TPDA-CZ-COF catalyst has been shown in Figure 5 [40]. The catalytic reduction involves the hydrolysis of NaBH_4_, generating active hydrogen species. In an alkaline medium, *p*-nitrophenol is converted into *p*-nitrophenolate ions, which are subsequently adsorbed on the catalyst surface and undergo hydrogenation, as shown in Figure 5b. This process leads to the formation of *p*-aminophenol as the final product [40].

The study by Zhang et al. prepared Ag@Co-MOF to enhance the conversion rate of p-NP. The study utilized novel photocatalyst for the reduction of p-NP using the hydrothermal method. AgNPs were incorporated on the surface of Co-MOF, which accelerated the photogenerated carrier transport. The incorporation of AgNPs also prevented the recombination of electrons and holes, resulting in degradation within 240 min with a removal rate of 92.7% for p-NP. The pore structure of Co-MOF enhances the adsorption of Ag; however, saturation occurs on the surface, decreasing the degradation efficiency [60].

Upon light excitation, electrons were shifted from the CB to the VB and are transferred to Ag^+^, forming metallic Ag deposited on the Co-MOF surface. The accumulated electrons react with oxygen to generate ·O_2_^−^ radicals, while holes in the valence band either directly oxidize p-NP or react with OH^−^ to form ·OH radicals, further enhancing degradation, as shown in Figure 5a. The catalyst retained good stability for up to five cycles, maintaining 85% degradation efficiency, with a visible-light degradation rate constant of 0.0095 min^−1^.

The study by Oueslati et al. prepared AuNPs derived from *Salvia officinalis* (SO) extract for the conversion of p-NP and MB. The study found that polyphenols extracted from SO act as capping agents on the catalyst surface during the reduction of p-NP and MB. Moreover, AuNPs act as catalysts to increase the conversion rate of MB and p-NP, as the addition of the prepared AuNPs in the presence of NaBH_4_ enhances the conversion efficiency. The AuNPs act as nanocatalysts and convert MB into leuomethylene blue (L-MB), and similarly, the decrease in the adsorption band shows the formation of p-AP [61]. In another study by Kale et al., lemongrass-derived CuNPs were taken into consideration for the reduction of p-NP. The study found that the prepared CuNPs were 2.1 nm in size and synthesized under controlled conditions using lemongrass extract as a reducing and capping agent [62]. In another study, Albukhari et al. developed AgNPs@CP for the reduction of nitrophenol. The AgNPs were prepared using *Duranta erecta* leaves, which act as a reducing agent. The silver nanocomposite was prepared using cellulose acetate filter paper and titanium dioxide (Ag@TiO_2_) via a biological route. After the addition of p-NP, the position of the peak shifted from 317 nm to 402 nm. The study found that without any active catalyst, sodium borohydride alone cannot reduce nitrophenol. After the addition of the catalyst, the nitrophenolate ions started decreasing. Furthermore, with the decrease in the peak, the formation of a new peak corresponding to p-AP takes place [63].

Zavala et al. prepared AuNPs using an eco-friendly technique for the conversion of p-NP. Zavala and his co-workers utilized *Pithecellobium dulce* (P. dulce) leaf extracts, which contain various phytoconstituents including flavonoids and polyphenols that act as stabilizing agents. These bioactive components prevent the aggregation of NPs by maintaining their dispersion. The biomolecules present in the plant extract can interact electronically with the Au surface, slightly affecting the adsorption performance and strength of both p-NP and BH_4_^−^ ions. These types of synergistic interactions are unique in that surface-bound phytochemicals and metal NPs enhance the catalytic performance. Within 25 min, complete reduction of p-NP occurs using nanostructures of size 12 nm, with a removal rate of up to 98% [64]. Also, Swain et al. employed s green method to synthesize N, S-doped CDs from Giloy stem to enhance the detection of p-NP. The prepared N,S-doped CDs exhibited high fluorescence, prepared using Giloy stem extract. In this study, the authors targeted pollutants including p-NP and Congo red, with reported LOD of 380 nM for p-NP and 62 nM for Congo red dye. The fluorescence quenching occurring in N, S-CDs was due to the inner filter effect, with a recovery range from 93.88 to 103.45% in real water samples. The quenching mechanism was investigated by observing the overlap between emission and excitation spectra of the prepared material. The partial overlap of N,S- CDs spectra with the absorbance spectra of p-NP reveals fluorescence quenching due to IE and FRET [65]. Table 5 highlights the comparison of already reported studies on pollutant degradation, including their synthesis methods, precursors, reduction time, efficiency, size, and catalyst reusability.

Tan et al. reported the preparation of AgNPs using *Ceratonia siliqua* pod aqueous extract for the removal of toxic pollutants. The prepared NPs exhibited a negative charge on the surface, which indicates good stability. TEM analysis revealed the average size of the NPs to be 33–43 nm with spherical morphology. The study observed the catalytic performance of AgNPs for NaBH_4_-assisted reduction of p-NP, o-aminonitrobenzne, 2,4-dinitophenol, and 1,2-diamino-4-nitrobenze under ambient conditions. The study highlights that the conversion of aminobenzene was reached within 1 min. The mechanism of the study exhibits adsorption of both borohydride and nitroaromatic molecules onto the AgNP surface, forming Ag-H species. The stepwise H-transfer to -No_2_ groups leads to their reduction to -NH_2_ groups. These finding shows the effectiveness of using carob extract as a capping agent during the production of AgNPs [66].

**Table 5:** Comparison of reported studies on pollutant degradation, including their synthesis methods, precursors, reduction time, efficiency, size, and catalyst reusability.

S/N	Catalyst	Method	Natural Precursor	Reduction Time (Minutes)	Pollutant	Rate Constant (min^−1^)	Size (nm)	Conversion Efficiency (%)	Surface Area (m^2^g^−1^)	Reusability (Cycles)	Ref.
1.	Au@TPDA-CZ-COF	Solvothermal Schiff-base condensation	Electronic waste	5	p-NP	0.965		99.24	587	5	[40]
2.	Ag Fe_3_O_4_/ATO	Hydrothermal		2.67	p-NP	1.048	55		-	5	[67]
3.	Magnetic hybrid adsorbent	Co-precipitation	Coconut mesocarp sawdust	0.72–0.93	p-NP		17–20	99.9	356.931	3	[41]
4.	Ag@ CAF; Ag@TiO_2_	Plant-extract-assisted impregnation	Duranta erecta leaves	12	p-NP	0.174; 0.0648	25	95	-	5	[63]
5.	Ag_2_S NPs	Green synthesis	Lemon citrus	420	MB	0.0013	17–23	80	-	-	[46]
6.	La_2_O_3_NPs	Green synthesis	Cymbopogoncitratus	180	MB	0.492	20–50	92.43	-	-	[68]
7.	ZnO NPs	Bio-reduction	Musa Paradisiaca plant	30	MB	0.08	15–25	91	-	3	[69]
8.	ZnO-NPs	Biogenic synthesis	Justicia adhatoda	180	p-NP	0.245		99.8	154.17	5	[53]
9.	Co_3_Fe_7_/CoFe_2_O_4_	In situ calcination	Citric acid	50	p-NP	0.031	5	79	517.58	5	[70]
10.	ZnO@henna	Green synthesis	Kaffir lime extract	120	p-NP		41.40	93	106.41	4	[71]
11.	NiFe_2_O_4_/poly (aniline-co-o-toluidine	Green synthesis	PEG	60	p-NP	5.44 × 10^−4^	32–68	99	-	4	[55]
12.	ZnO NPs	Sol–gel method		420	MB		31.09	72.3	-	-	[49]
13.	AuNPs	Green synthesis	Korean red ginseng	6.67	MB	0.128–1.009	2.4–5.1		-	-	[44]
14.	Fe_3_O_4_-NPs	Green synthesis	Tinospora cordifolia	60	MB			88	-	5	[45]
15.	ZnO NPs	Co-precipitation	Abroma augusta	150	MB		12	91	-	-	[47]
16.	AuNPs	Green synthesis	Pithecellobiumdulce	25	p-NP	0.49 ± 0.18	12	98	-	-	[64]
17.	AgNPs	Green synthesis	Merremia quinquefolia	240	MB		14	94.89	-	--	[48]
18.	NiO NPs	Green synthesis	Cotton	14	p-NP	0.494	32		-	10	[57]
19.	Au NPs, Ag NPs	Green method	Camellia sinensis	20; 60	p-NP	0.009–0.054; 0.03–0.18	53.92; 18.50		-	-	[56]

### 5.3. Experimental Parameters

#### 5.3.1. Catalyst Dosage

The study by Karki et al. found that an increase in catalyst dosage enhances the removal rate. The study varied the sample dosage from 0.6 g/L, 0.9 g/L, 1.2 g/L and 1.5 g/L at different time intervals. The reduction rate of PNP at 1.2 g/L catalytic dosage was higher than the others. The removal rate reached 99.62% within 160 s. Further increasing the dosage to 1.5 g/L resulted in a faster removal rate within 80 s, which was difficult to interpret [67]. Also, the study by Zhang et al. varied the catalyst dosage from 0.5, 0.75, 1.00, and 1.25 g/L to observe the optimal dosage. The degradation efficiency at 1.00 g/L was higher compared to the others, attributed to the presence of more active sites. However, a further increase in dosage led to decrease in degradation efficiency due to an increase in the turbidity of the solution [60]. Similarly, varying the dosages from 25 to 75 μL of the prepared catalyst under pH 8 was carried out to obtain the optimum value. The study revealed that at higher dosages, aggregation occurred. The degradation efficiency increased with increasing catalyst dosage, as reflected by the progressive rise in the apparent rate constant (Kapp). This indicates that a higher catalyst amount provides more active sites, thereby accelerating the reduction of both PNP and MB. However, further increase beyond the optimum may lead to particle aggregation, as suggested by the turbidity observed at higher doses [9]. In the study by Stambouli et al., the prepared NiFe_2_O_4_/PAOT catalyst was used for the effective conversion of p-NP to the less harmful p-AP. The authors optimized the catalyst dosage to obtain the optimal dose at which degradation efficiency was high. The evaluation was conducted by varying the catalyst amount from 5 mg, 10 mg, and 20 mg, respectively. The study reported that catalyst dosage enhances reduction efficiency; with an increase in dosage, there is an improvement in reduction efficiency, highlighting the importance of optimizing catalyst dosage [55]. The study by Eisa found that the rate of the catalytic degradation reaction is highly related to the dose of the catalyst (NiO@cotton). The study revealed that increasing the dose of the catalyst from 0.01 to 0.07 g increased the rate constant. The increase in the reaction rate constant was related to the availability of more active sites on the surface of the cotton matrix [57].

#### 5.3.2. Temperature Effect

The study by Stambouli et al. reported two different temperatures, 37 °C and 50 °C, to investigate the catalytic efficiency at different temperatures. The prepared NiFe_2_O_4_/POAT catalyst showed a lower reduction rate at 37 °C than at 50 °C. This shows that an increase in temperature promotes the electron transfer process, thereby enhancing catalytic performance [55]. In another study, Laghrib et al. prepared TiO_2_ NPs for the catalytic reduction of MB. The study found that crystalline size increases with increasing temperature. The temperature was varied from 300 to 700 °C to observe the NM performance. In an acidic solution, the crystal size expanded from about 23 nm at 400 °C to 56 nm at 600 °C, while in a neutral medium, it increased from 25 nm at 300 °C to 84 nm at 700 °C. The catalytic activity, electron transfer efficiency, and surface characteristics of the nanomaterials are all directly impacted by this rise in particle size and crystallinity [72]. In another study, Upadhyay et al. prepared CuO NPs for the reduction of methylene blue. The study varied the temperature and found an optimal temperature of 60 °C. The study reported that very high temperature ranges may result in thermal deactivation [73].

#### 5.3.3. pH Effect

The pH effect was assessed by analyzing the pH range from 5.0 to 10.0 for PNP. The study found the optimal pH of 8–9 for PNP and MB. The pH range from 7 to 9 was found to be optimal for the reduction of pollutants. Moreover, the study revealed that hydroxyl groups compete with hydride ions at a pH of less than 9, in which the transfer of hydride ions is inhibited [9]. Similarly, Eisa et al. showed that an increase in pH value from 6 to 10 increases the pH effect. In this respect, one can conclude that an alkaline medium accelerates the hydrolysis of NaBH_4_, which provides reactive hydrogen for the reduction of p-NP into p-AP [57]. In another study, Upadhyay et al. tested the pH range from 7 to 11 during the synthesis of CuO NPs. The study found the maximum absorption intensity at pH 11, which starts decreasing at pH 12. The authors states that with an increase in pH, absorption intensity also increases [73].

## 6. Conclusions and Future Perspectives

In past decades, green synthesis of NMs has represented an emerging area of research with significant advancements. The utilization of green precursors provides an eco-friendly alternative to conventional chemical approaches. This process not only eliminates the requirement for toxic chemicals but also reduces stress on the environment. In contrast to chemical precursors, green precursors offer organic compounds that act as both stabilizing and reducing agents. Due to their cost-effectiveness, low energy requirement, and unique physicochemical properties, green precursors are more frequently utilized. This review explores various types of materials, including (i) polymer-based NPs; (ii) MNPs; (iii) metal-oxide NPs; and (iv) carbon-based materials, for the efficient reduction of pollutants like p-NP and MB. This comprehensive review covers recent advancements in the conversion of p-NP and MB using green precursor-derived NMs. Various studies have reported that the prepared catalysts exhibit promising potential for wastewater treatment. Moreover, it has been found that NMs prepared using different precursors and synthesis methods exhibit varying efficiencies and recyclability. The lack of reproducibility occurs due to different extraction conditions, precursor composition, and molecular content. Green precursors contain different types of organic compounds (flavonoids, phenolics, and proteins), which differ depending on the season, precursor source, and extraction procedures. This variability in natural precursors affects the size, shape, morphology, and catalytic performance of NMs. This difference restricts their scalability and hinders their standardization for industrial applications. Therefore, researchers are still struggling to identify an efficient synthesis method and a suitable type of precursor for high efficiency. Moreover, various studies have achieved high efficiency under varying optimal required temperature and pH conditions. However, this affects the real-time applicability of prepared NMs. Under real-world scenarios, issues of selectivity and operating conditions remain significant challenges.

Furthermore, the yield of prepared NMs using green precursors is still low, which remains an active area of research. No doubt that green-prepared NMs exhibit efficiency and environmental compatibility, but concerns related to catalyst leaching still require investigation. Metal NP-based catalysts undergo oxidation, aggregation, and leaching during repeated usage, which reduces their reusability and produces secondary contaminants. Operational challenges also arise from the recovery and recyclability of NMs, especially for non-magnetic NPs that need supplementary separation procedures. Furthermore, more research is necessary to completely understand the molecular catalytic mechanisms, such as surface contacts, electron transfer channels, and active site stability. Future research should focus on the development of defined protocols to ensure reproducibility. Advanced methods like material modification, composite formation, and incorporation of foreign materials can enhance catalytic efficiency. More interest should be given to understanding mechanistic insights through advanced characterization techniques. Additionally, focus should be placed on synthesizing NMs capable of degrading different types of pollutants, as wastewater is contaminated with multiple pollutants, and the effects of other competing pollutants in the water still need to be explored.

There is an urgent need to scale up the green synthesis processes for the treatment of real industrial wastewater. It is also important to assess the regulatory aspects of green nanocatalysts and evaluate their life cycle impacts. Overall, the use of green precursors for the reduction of toxic pollutants holds strong potential for wastewater treatment and contributes significantly to environmental sustainability.

## Figures and Tables

**Table 1:** Comparison of the current review with recently published review articles on the green synthesis of NPs for the catalytic reduction of MB and p-NP.

Title	Synthesis Methods and Physicochemical Properties	Green Precursors	Catalytic Mechanism	Catalytic Reduction of Para-Nitrophenol	Catalytic Reduction of Methylene Blue	Catalytic Reduction of Both Methylene Blue and Nitrophenol	Ref.
Nanostructures embedded on porous materials for the catalytic reduction of nitrophenols: a concise review	√	×	√	√	×	×	[28]
A comprehensive review on biogenic synthesis of bimetallic NPs and their application as catalytic reduction of 4-NP	√	√	√	√	×	×	[29]
Waste-derived 0D NMs for the catalysis reduction of p-NP: A technological progress and developments	√	√	√	√	×	×	[30]
Critical review on development of MB degradation by wet catalytic methods	√	×	√	×	√	×	[31]
An expanded review of green synthesis NPs for the removal of industrial effluents, specifically MB	×	√	√	×	√	×	[32]
Photocatalytic degradation of MB: performance, mechanism, and perspectives	×	×	√	×	√	×	[33]
a review of the recent advances of CDs in the application of photocatalytic Degradation of MB dye and their future prospects	×	√	√	×	√	×	[34]
A review on green synthesis of AgNPs using plant extracts: a multifaceted approach in photocatalysis, environmental remediation, and biomedicine	√	√	√	×	√	×	[35]
A compendium on the eco-sustainable biosynthesis of PdNPs and their new avenues towards environmental applications	√	√	√	√	×	×	[36]
Eco-friendly synthesis of green nanoparticles for the catalytic reduction of NP and MB: a review	√	√	√	√	√	√	This work

√ indicates covered; × indicates not covered.

**Table 2:** Physicochemical properties of NMs for the removal of MB and p-NP.

Properties	Range	Characterization Techniques	Reduction Role
Average Particle Size	<100 nm (Commonly 5–50 nm)	TEM, SEM	The smaller the size, the greater the number of active sites
Morphology	Spherical, rod-like, flower-like	SEM, TEM	Controls diffusion pathways
Pore Diameter	2–50 nm	BJH	Enhance access to active sites
Pore Volume	0.05–0.6 cm^3^ g^−1^	BET	Increases mass transfer rate
Surface Area	10–500 m^2^g^−1^	BET	Provides active sites, enhancing reduction efficiency
Zeta Potential	−40 to +35 mV	Zetasiezer	Reveals stability
Thermal Stability	Stable up to 500 °C	TGA	Facilitates reusable capacity
Hydrophilicity	High	Contact Angle	Enhances aqueous interaction
Chemical Stability	Water stable	Leaching Tests	Prevention of catalyst degradation
Recyclability	3–10 cycles	Batch Tests	Practical applicability
Leaching of Metal Ions	<5%	ICP-OES	Eco-friendly
Catalytic Rate Constant	0.01–1.5 min^−1^	UV-Vis Kinetics	Measures catalytic efficiency
Environmental Stability	High	Stability tests	Long-term usage

**Table 3:** Physicochemical properties of methylene blue.

Chemical Structure	Molecular Formula	Molecular Weight	Maximum Absorption Wavelength	Structure Type
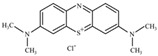	C_16_H_18_ClN_3_S	319.85 g·mol^−1^	660–665 nm	Aromatic heterocyclic with planar conjugated system

**Table 4:** Physicochemical properties of p-nitrophenol.

Chemical Structure	Molecular Formula	Molecular Weight	Maximum Absorption Wavelength	Structure Type
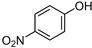	C_6_H_5_NO_3_	139.11 g·mol^−1^	317–400 nm	Aromatic benzene ring with –OH and –NO_2_ groups

## Data Availability

No new data were created or analyzed in this study.

## Abbreviations

The following abbreviations are used in this manuscript:

NMs	Nanomaterials
NPs	Nanoparticles
p-NP	Para-nitrophenol
p-AP	Para-aminophenol
VB, CB	Valence band, conduction band
MB	Methylene blue
ROS	Reactive oxygen species
AgNPs@CP	Silver nanoparticles @ cellulose polymer
AuNPs	Gold nanoparticles
A.S	*Aspergillus* sp.
(NiFe_2_O_4_)/poly(aniline-co-o-toluidine)	Nickel ferrite/poly(aniline-co-o-toluidine)
ZnO; NiO	Zinc oxide; nickel oxide
MNPs	Metal nanoparticles
SDG’s	Sustainable Development Goals
PdNPs	Palladium nanoparticles
AgNPs	Silver nanoparticles
CDs	Carbon dots
